# Characteristics and predictors of readiness to quit among emergency medical patients presenting with respiratory symptoms

**DOI:** 10.1186/1865-1380-4-24

**Published:** 2011-06-06

**Authors:** Beth C Bock, Ernestine Jennings, Bruce M Becker, Robert Partridge, Raymond S Niaura

**Affiliations:** 1 Centers for Behavioral and Preventive Medicine, The Miriam Hospital, 167 Point Street, Providence, RI 02903, USA; 2 Department of Emergency Medicine, Rhode Island Hospital, 55 Eddy Street, Providence, RI 02903, USA; 3 Department of Community Health, Alpert School of Medicine at Brown University, 1 Hoppin Street, Providence, RI 02903, USA; 4 Schroeder Institute for Tobacco Research and Policy Studies, American Legacy Foundation, 1724 Massachusetts Avenue NW, Washington, DC, 20036, USA

## Abstract

**Purpose:**

To examine behavioral factors that lead patients to consider quitting smoking and features associated with readiness to quit among adults who are seeking treatment in the emergency department (ED) for respiratory symptoms.

**Methods:**

A toal of 665 adult smokers seeking treatment in an ED for respiratory symptoms and respiratory illness answered survey questions during the ED visit.

**Results:**

Patients self-reported "readiness to quit" was broadly distributed among this patient population. Patients with COPD, pneumonia or asthma perceived higher risks from smoking than other patients with respiratory complaints. Over half of all participants had scores indicative of depression. Regression analysis showed that prior efforts to quit, confidence, perceived importance of quitting and decisional balance were each significantly predictive of readiness to quit, accounting for 40% of the variance.

**Conclusions:**

While many of these patients appear unaware of the connection between their symptoms and their smoking, patients with diagnosed chronic respiratory illness perceived higher risks from their smoking. In patients who do not perceive these risks, physician intervention may increase perceived risk from smoking and perceived importance of quitting. Interventions designed for the ED setting targeting this patient population should consider screening for depressive symptoms and, when appropriate, making referrals for further evaluation and/or treatment. Medications that can help alleviate depression and withdrawal symptoms while quitting smoking, such as bupropion, may be particularly useful for this subset of patients, as depression is a substantial barrier to quitting.

## Introduction

Over 12 million visits each year are made to emergency departments for respiratory illness [1,2]. Chronic respiratory illnesses are among the most common chronic medical conditions in the US, affecting over 25 million adults [3,4]. All-cause mortality rates due to smoking have decreased since the 1960s; however, there has been a significant rise in morbidity and mortality from respiratory illness [5-7]. Two important contributors to this trend are the persistence of cigarette smoking and an increasing dependence upon crisis-oriented care among persons with chronic respiratory illness [8-10].

Cigarette smoking is the single most important risk factor for the development of acute and chronic respiratory illness, acute exacerbations of respiratory illness, and associated morbidity and mortality [11-15]. Among adults with respiratory illness, exposure to tobacco smoke increases the rate of acute episodes, ED visits, work absences and frequency of medication use [16]. Likewise, asthmatics who smoke show greater declines in lung function, worsening of respiratory symptoms and lower quality of life compared to non-smoking asthmatics [17,18]. Current data suggest that 50-80% of asthma-related deaths are preventable through improved self-management and changing risk behaviors like smoking [12].

Successful smoking cessation treatment has been linked to a persons' readiness to change their smoking behavior and a number of psychological and behavioral attributes associated with readiness to change [19]. The Transtheoretical Model (TTM) of Behavior Change defines a persons' readiness to change as a progression through the five stages: Precontemplation, Contemplation, Preparation, Action, and Maintenance [20]. The first stage, Precontemplation, encompasses individuals who are highly ambivalent about changing their behavior and who do not intend to take action toward behavior change in the next 6 months. In Contemplation, individuals recognize a problem exists and consider changing; however they are not yet committed. Individuals in the Preparation stage are convinced that the advantages of change outweigh the disadvantages and are ready to act within the next 30 days. The Action stage characterizes those who have successfully altered their behavior within the past 6 months, while "Maintenance" describes those who have maintained the new behavior for at least 6 months. Numerous studies have shown that additional behavioral and cognitive factors including decision-making, confidence and perceived risk [20-22] also change along with readiness. These stages provide a paradigm in which to view the change process, allowing clinicians to understand the progression and use motivational strategies to facilitate movement through the stages toward sustainable change [23,24].

We examined the psychological and behavioral factors that are relevant to smoking cessation among a population of adult men and women who presented to the ED with symptoms of respiratory illness. The results of this study have implications for the feasibility and design of smoking cessation interventions for the 5 million smokers treated in EDs each year.

## Methods

### Inclusion-exclusion criteria

Recruitment began after approval was obtained from the Institutional Review Board. Participants included adult men and women seeking treatment in the emergency department (ED) of a large, urban, teaching hospital for acute or chronic respiratory illness (including both upper and lower respiratory illness) or symptoms of respiratory illness. Lower respiratory symptoms included at least one of the following: cough, shortness of breath or wheeze. The diagnoses that these symptoms encompass included but were not limited to: pneumonia, asthma exacerbation, acute bronchitis, asthmatic bronchitis, exacerbation of chronic obstructive pulmonary disease and exacerbation of emphysema. Upper respiratory symptoms included at least one of the following: rhinorrhea, nasal stuffiness or sore throat. The diagnoses that these symptoms encompassed included but were not limited to: acute sinusitis, rhino-sinusitis, acute infectious rhinitis, pharyngitis, laryngitis, tracheitis and uvulitis. Other eligibility criteria specified that a participant must: (1) be at least 18 years of age, (2) be a current, regular smoker (smoke daily for the past 3 months), (3) speak English or Spanish, (4) be reachable by telephone and (5) agree to participate in the study and be available for follow-up assessments.

### Measures

#### Motivation to quit smoking

Motivation to quit smoking was assessed using the Contemplation Ladder [25], the stages of change questionnaire [26] and a single item on which participants rated on a 1-10 scale how ready they were to quit smoking ("Readiness"). The Ladder is a continuous measure of motivation to change smoking behavior that uses a 10-point scale with responses ranging from 1 = "I have decided to continue smoking" to 10 = "I have already quit smoking." Validity studies have demonstrated that the Ladder is associated with cognitive and behavioral indices of readiness to consider smoking cessation (e.g., intention to quit, nicotine dependence) and performs as well or better than the staging algorithm in predicting smoking rate, quit attempts and cessation [25,27,28].

#### Smoking Decisional Balance Scale

The Decisional Balance Scale (short form) is a six-item measure of the perceived benefits ("pros") and drawbacks ("cons") associated with smoking. Participants endorse agreement with each item on a 5-point scale (1 = not at all; 5 = very much). The scale is divided into Pros and Cons subscales, both of which had high internal validity in prior studies (alpha = 0.88 and 0.89, respectively) [29]. The subscale scores are used to gauge the degree to which smoking remains important for the individual smoker.

#### Smoking temptations and confidence in quitting

We used the short form of the Situational Temptation Inventory (STI) [30,31]. Participants use this nine-item measure to report how tempted to smoke they would feel under a variety of circumstances. The STI has three subscales that correspond to Habit, Social and Mood-related triggers for smoking. Confidence was assessed using a single question that asked participants "if you decided to quit smoking, how confident are you that you could quit?" Participants marked their answers on a 1-10 scale from 1 "not confident" to 10 "very confident."

#### Risk perception

Participants' perception of health risk due to smoking was assessed using five items validated in prior research [32,33]. Three items assessed the degree to which (1) smoking has affected their overall health, (2) their respiratory symptoms are related to their smoking and (3) quitting smoking would improve health. Three other items assessed the participant's perceptions of their health status relative to other smokers their own age, and whether or not they had (in the past) or currently have an illness or condition caused or made worse by smoking.

#### Depressive symptoms

Symptoms of depression were assessed using the ten-item Center for Epidemiologic Studies Depression Scale CES-D [34]. Use of a brief depression measure is important given the time limitations inherent in approaching patients in the ED. Additionally, symptoms of depression, measured via the CES-D, have been significantly associated with current smoking status and difficulty quitting among Hispanics and in the general population [35-37].

#### Fagerstrom Test for Nicotine Dependence

This instrument [38] is a widely used measure of nicotine dependence. It has six items assessing amount of smoking, the time of the first cigarette after waking, smoking or not smoking in case of illness, ability to refrain from smoking in non-smoking place, reporting or not reporting the first cigarette of the day as the most difficult to give up, and smoking or not smoking more heavily in the morning. A score of 6 or higher identifies participants with high nicotine dependence.

#### Sociodemographic, smoking history and medical utilization

Sociodemographic and smoking history data were collected by questionnaire and included: age, sex, marital status, ethnicity, employment, occupation, education, income, current smoking rate, years smoked, previous quit attempts and prior use of medications to quit smoking. Participants indicated the number of medical visits (including ED visits, hospitalizations and primary care visits) in the past year, and responded to questions about whether their personal physician had ever advised them to quit smoking, and whether the ED physician (current visit) had asked about their smoking or advised them to quit. Information obtained at baseline from the ED patient triage roster was used to determine the participant's presenting chief complaint.

### Procedure

Smokers presenting to the ED for treatment of respiratory symptoms were identified by a trained research associate (RA) who routinely reviewed the admissions roster kept at the triage desk. This roster included the name, presenting complaint and location within the ED of all patients admitted to the ED. The typical duration of a patient's stay in the ED is 3-4 h, providing ample time for case identification and intervention. Patients were approached by the RA who explained the study, determined interest, reviewed inclusion/exclusion criteria and obtained written, informed consent. The recruitment strategy utilized an approach that initially offered the patient an opportunity to discuss their current illness, and their satisfaction with their experience thus far in the ED, gradually narrowing to the identification of smoking status. This topic-narrowing approach was used to maximize representation in our sample of smokers who are less motivated to quit, and who might therefore be less likely to enroll in a study about smoking cessation.

After providing written consent, participants completed the questionnaires assessing socio-demographic information, smoking history (e.g., years, quit attempts, etc.), motivation to quit, confidence in remaining abstinent, reasons to continue to smoke (pros), barriers to quitting (cons) and perceived vulnerability to smoking-related illness. Time to completion of the study introduction, consent procedures and questionnaire was no more than 15 min.

### Statistical Analyses

Descriptive data are presented in terms of actual number (*n*) and percent of the sample population along with means for groups and standard deviations (SD). Pearson correlations were used to test the association between continuous variables. One-way ANOVAs and chi square analyses were used to examine differences between groups (e.g., gender). A single regression analysis was conducted to examine predictors of readiness to quit smoking. All *p *values reported are two-tailed, and all statistical analyses were performed using the Statistical Package for Social Sciences, version 13.0 (SPSS, Inc. Chicago, IL).

## Results

### Participants and smoking patterns

RAs reviewed the admission logs identifying a total of 4,002 patients who were admitted to the emergency department with respiratory symptoms. Of these, 36.8% (*n *= 1,619) were non-smokers, 10.2% (*n *= 448) had been triaged to the ICU, 3.5% (*n *= 158) were unavailable for recruitment (e.g., busy with tests, therapy or physician visits) and 18.4% (*n *= 809) did not meet eligibility criteria. Of those eligible for the study, 303 (31%) refused participation. The RAs recruited the remaining 665 individuals into the study.

A total of 277 men and 388 women met criteria for the study and completed informed consent. Average age was 37.5 years (range: 18 to 80 years). About half (52%) were non-Hispanic white, and 72% had 12 years or less of formal education (Table 1). The most common presenting complaint was shortness of breath (25.8%), followed by cough (20%) and sore throat (13.9%). Overall 69.7% of participants had a lower respiratory complaint. Over 90% of participants had at least one prior ED visit in the past year. Table 1 lists the participants' demographic characteristics and past year's medical utilization. Table 2 lists presenting complaints.

**Table 1:** Demographic characteristics and medical information (*N *= 665)

Variable	Number of patients (%)
**Gender**	
Male	277 (42%)
Female	388 (58%)
**Racial/ethnic group**	
Caucasian	341 (52%)
Hispanic	114 (17%)
Black	127 (19%)
Asian	5 (1%)
American Indian	20 (3%)
Mixed ethnicity	52 (8%)
**Years of education**	
12 years or less	478 (72%)
Some college	152 (23%)
College graduate	27 (4%)
Post graduate	6 (1%)
**Employment status**	
Full-time	36%
Part-time	10%
Unemployed	26.4%
Disabled	20.5%
Retired	4.8%
Student/volunteer/other	2.3%
**Marital status**	
Single	301 (46%)
Living with significant other	89 (13%)
Married	126 (19%)
Divorced or separated	117 (18%)
Widowed	29 (4%)
**Total household income**	
Under 10,000	187 (29%)
10,000-19,999	124 (19%)
20,000-29,999	85 (13%)
30,000-39,999	46 (7%)
40,000-49,999	23 (4%)
50,000 and over	39 (6%)
	**Mean (SD)**
**Smokers in household**	2 (1.8)
In past year:	
Number of visits to doctor	6.52 (15.4)
Number of visits to ED	4.14 (11.7)
Number of hospitalizations	1.19 (6.8
Number of days in hospital	3.6 (12.4)

**Table 2:** Presenting complaint

	Frequency	Percent
Shortness of breath*	173	25.8

Cough*	134	20.0

Sore throat	93	13.9

Asthma*	76	11.3

Bronchitis, chest congestion*	37	5.5

Pneumonia*	26	3.9

Ear infection, earache	22	3.3

Cold symptoms	21	3.1

Sinus infection	13	1.9

Wheezing*	11	1.6

COPD*	10	1.5

Other	54	8.0

*Asterisk denotes presenting complaints/diagnoses counted as lower respiratory illness in the analyses.

Participants smoked an average of 15.3 (SD = 10.9) cigarettes a day and reported an average of three serious (24 h or longer) quit attempts in the past year. Twenty-three percent of participants said that they had quit smoking for 1 full year or longer at some time in the past. The average score on the nicotine dependence scale was 4.78 (SD = 2.3). Not surprisingly, higher nicotine dependence scores were positively correlated with the number of cigarettes currently smoked (*r *= 0.621, *p *< 0.001).

### Motivation/readiness to quit smoking

The average score on the Contemplation Ladder was 6.4 (SD = 1.9, range = 1-10). Average score on the single-item Readiness measure was 6.1 (SD = 2.7, range = 1-10). The distribution of scores on the Stages of Change assessment was 61.6% in "Preparation" (planning to quit within 30 days), 27.9% in "Contemplation (planning to quit within 6 months), and 10.4% in "Pre-contemplation" (not planning to quit). Since all three measures of motivation to quit smoking were well correlated with each other, we used the single item Readiness question as the measure of motivation for all additional analyses.

### Decisional balance, temptations, confidence for quitting and depression

As a group, the average score on the Pros subscale of the Decisional Balance measure score was not significantly different than the average Cons score (M = 10.32, SD = 2.6 versus M = 10.56, SD = 2.5), suggesting that individuals agreed at least somewhat with statements reflecting the advantages and the disadvantages of smoking. The combined STI score for all three subscales averaged 33.2 (range = 6-45; SD = 6.7), indicating that participants were moderately to very tempted to smoke in a variety of situations. The highest scores reflected temptations to smoke in emotional situations (M = 12.7, SD = 2.6), with social situations (M = 10.7, SD = 2.8) and habit-related temptations (M = 9.9, SD = 3.0) having lower scores. Scores on the Confidence measure averaged 5.2 (SD = 2.6) on a 1-10 scale. Nearly one-quarter (22%) of participants noted that they were only "slightly" or "not at all" confident, while 24% stated that they were "very" or "extremely" confident in their ability to quit smoking. Confidence was negatively correlated with both the number of cigarettes smoked per day (*r *= -0.128, *p *< 0.01) and with nicotine dependence (*r *= -0.13, *p *< 0.01).

On average, individuals presenting to the ED with respiratory symptoms endorsed relatively high levels of depressive symptoms on the CESD-10 (M = 13.59, SD = 6.7). Overall, 69% of participants (*n *= 458) had CES-D-10 scores equal to or greater than 10, which is indicative of depression [34,38]. Women had higher average CESD scores compared with men (14.1, SD = 6.9 vs. 12.8, SD = 6.0: F[1,663] = 10.8, *p *< 0.001). When participants were divided into two groups based on the score of 10 cutoff, those with lower depression scores had lower scores on the STI temptations measure (F[1,663] = 34.7, *p *< 0.001), lower nicotine dependence scores (F[1,663] = 12.05, *p *< 0.001) and higher risk perception (F[1,663] = 21.1, *p *< 0.001) compared to those with more depression symptoms, suggesting that those smokers who had lower depression scores perceived fewer barriers to quitting. However, there were no differences between those with higher and lower depression symptoms for confidence in ability to stop smoking or in Readiness to quit smoking.

### Risk perception

Total scores of the five risk perception items averaged 15.5 (range = 6-22, SD = 3.5). Over half (56%) of participants agreed that they currently had a disease or symptoms that had been "caused or made worse" by smoking. However, most (62%) believed their health was "about the same," "better" or "much better" than the average smoker their age. On questions of whether their illness might be related to their smoking and how much their health was affected by smoking, scores were evenly distributed across all answer categories (1-5 scale). However, over 86% of participants stated that quitting smoking could help their health "very much" or "quite a bit" (5 or 4 on a 1-5 scale).

Risk perception varied significantly by presenting complaint (F[11,591] = 2.9, *p *< 0.001). Post-hoc analyses showed that individuals with lower respiratory complaints had significantly higher risk perception scores than those with upper respiratory symptoms (F[11,603] = 2.91, *p *< 0.001) (Table 3). For example, 100% of patients with COPD endorsed "yes" in response to the question "Do you have symptoms of a disease or illness that is caused or made worse by smoking?", compared to two-thirds of those with asthma, bronchitis, shortness or breath, sinus infection or pneumonia, and approximately 50% of those with a cold, cough, wheezing or ear infection. Only 38% of individuals with a sore throat thought that their illness was adversely affected or caused by their smoking. These differences in proportions were significant (χ^2 ^(11) = 29.85, *p *< 0.002).

**Table 3:** Risk perception among patients with upper versus lower respiratory complaints

Risk perception questions	Upper	Lower		Significance
	*% (n)*	*% (n)*		*Chi-square*

1) Past illness caused or made worse by smoking? (yes)	75.6% (235)	24.4% (76)	51.2%	χ^2 ^= 9.26*

2) Do you now have symptoms of an illness cause or made worse by smoking? (yes)	74.7% (266)	25.3% (90)	49.4%	χ^2 ^= 9.41*

	*Mean (SD)*	*Mean (SD)*	*Difference*	*95% CI*

3) To what degree has smoking affected your health?	3.2 (1.2)	3.5 (1.1)	0.289	0.094-0.487*

4) To what degree are your current symptoms related to your smoking?	2.7 (1.4)	3.1 (1.3)	0.431	0.201-0.662*

5) To what degree would quitting smoking improve your health?	4.5 (0.8)	4.4 (0.9)	0.074	0.076-0.225

6) How is your health compared to other smokers your own age?	3.2 (1.0)	3.3 (1.1)	0.083	0.092-0.259

Overall risk perception	14.9 (3.3)	15.8 (3.4)	0.88	0.31-1.4*

*Indicates differences significant at *p *< 0.01

### Physician intervention

Overall, 80% of participants reported that they were asked their smoking status, but only 38.9% reported receiving direct advice to quit smoking while in the ED. Patients with lower respiratory illness (69.7% of participants) were significantly more likely to be asked about their smoking status (OR = 1.41; 95% CI = 1.19-1.67), but were not more likely to be advised to quit compared to participants with upper respiratory symptoms. Participants who received advice to quit smoking from the ED physician perceived greater risk to their health from continued smoking (F[1,636] = 10.7, *p *< 0.001) and were more ready to quit smoking (F[1,636] = 4.2, *p *< 0.05) compared to those not advised to quit. Perceived risk was not associated with medical utilization in the past year or with the ED physician simply asking about smoking status.

### Predictors of readiness to quit

Correlational analyses showed that readiness to quit smoking was significantly associated with medical utilization (number of ED visits and days in hospital), ED physician advice to quit smoking, perception of health risk from smoking, nicotine dependence, and the perceived benefits and hazards (Decisional balance "pros" and "cons") related to smoking (all correlations significant at *p *< 0.01). Correlations with Readiness to quit smoking are presented in Table 4.

**Table 4:** Correlations between variables and the single-item Readiness to Change scores (*N *= 665)

Correlations		
	Readiness to quit	
	Pearson correlation	Significance (2-tailed)
**Medical utilization in past year**		
Number of doctor's office visits	0.06	ns
Number of ED visits	0.10	0.06
Number of hospitalizations	0.04	ns
Number of days in hospital	0.11	0.07
**Physician intervention**		
Did the physician in the ED ask you about your smoking?	0.022	ns
Did the physician in the ED advise you to quit smoking?	-0.114	0.004
**Risk perception**		
Total risk perception score	0.222	<0.001
Do you have symptoms of an illness that is caused or made worse by smoking?	0.175	<0.001
How much has smoking affected your overall health?	0.207	<0.001
How much could quitting smoking help your health?	0.233	<0.001
**Other Variables**		
Decisional balance (Pros of continued smoking)	-0.19	<0.001
Decisional balance (Cons of continued smoking)	0.15	<0.001
Nicotine dependence	-0.131	<0.001

To determine which variables were most predictive of readiness to quit, all the above variables were entered into a linear regression analysis. Four items were significantly predictive of readiness to quit: Risk perception (beta = 0.18, t = 3.73, *p *< 0.001); Number of days in hospital in past year (beta = 0.10, t = 2.14, *p *< 0.05); and Decisional Balance Pros (beta = -0.17, t = 4.48, *p *< 0.001), and Cons (beta = -0.10, t = 2.56, *p *= 0.01). Combined, these items accounted for 40% of the variance in readiness to quit.

## Discussion

Results of this study indicate that a significant proportion of patients who are seeking emergency medical treatment for respiratory symptoms are smokers who may benefit from a smoking cessation intervention. Prior research has demonstrated that 20-30% of all ED patients [39] and up to 48% of patients with respiratory illness [40] are smokers. While ED patients in this and other studies have expressed interest in quitting smoking [39,41], our data appear to indicate that patients being treated in the ED for respiratory symptoms and illness may be more highly motivated to quit than general samples of ED patients. Other studies have documented that approximately 12% of ED patients who smoke endorse high levels of readiness to quit smoking [42,43]. In the current study of emergency respiratory patients, 23% of participants were planning to quit in the next 30 days, and an additional 19% endorsed responses indicating higher levels of motivation to quit. While the measures used to assess motivation were not identical between these studies, these data suggest that ED patients with respiratory symptoms may be more highly motivated to quit than the general population of ED patients.

Although all our participants had respiratory symptoms, only slightly more than half agreed that they had symptoms of a disease or illness that was caused or made worse by smoking, and a majority believed their health was the same or better than other smokers their own age. They expressed this optimistic belief, in spite of the fact that over half reported previous ED visits in the past year, and nearly one-third reported being hospitalized in the past year. These results seem to suggest that while emergency respiratory patients are motivated to quit smoking at the time of their ED visit, many may not be aware of the extent of the connection between their symptoms and their smoking. The concept of perceived risk is central to many important theoretical models of health behavior change including the Health Belief Model [44], Protection Motivation Theory [45], the Precaution Adoption Model [46] and the Theory of Reasoned Action [47]. Personalized information about health risk can be used to significantly alter patients' risk perception [48,49]. Interventions that are targeted to ED patients with respiratory symptoms may be more effective if they are developed using theoretical models, such as the Precaution Adoption Model [46], that incorporate the construct of perceived risk into the intervention.

Levels of depressive symptoms as measured by the CESD-10 were high in this population. Scores at or above 10 points on the CESD-10 are considered indicative of depression [34,47], and nearly 70% of our participants scored at or above that level. The mean CESD-10 score of 13.59 observed in our sample is equivalent to the CESD score observed by Almeida and Pfaff [50] in their sample of older general practice patients who smoked (M = 13.1). The fact that over half of our sample exhibited depressive symptoms suggests that ED patients with respiratory symptoms who smoke may benefit from interventions that include components that are designed to reduce depressive symptoms as depression inhibits the success of quit attempts in smokers.

Individuals with differing presenting complaints also differed in the degree to which they perceived health risk from smoking. Not surprisingly, those with lower respiratory illnesses including chronic conditions such as COPD and asthma were more likely to perceive a link between their smoking and their symptoms when compared to those with more transient conditions (e.g., sinus infection, sore throat). There are a number of possible explanations for the heightened awareness of the health risk from smoking among those with COPD or asthma. The presence of shortness of breath and other life-threatening respiratory symptoms experienced regularly by those suffering from these chronic illnesses may make breathing and any activities associated with breath more salient for these individuals. Alternatively many smoking patients with upper respiratory infections, which are common in the non-smoking general population, may not feel smoking caused or affected their acute illness. Patients with chronic medical illness have an ongoing and repeated exposure to health care providers and health care settings; they take medications regularly and have often been treated in EDs and inpatient units specifically for their respiratory illness. Thus, their awareness of health risk and their fear of negative consequences from their condition may be intensified as compared to those without these chronic illnesses. Furthermore, the optimistic bias and denial of associated risk commonly expressed by those smoking participants who do not have these conditions may be blunted. Surprisingly, though, neither the overall general medical utilization of our patient population nor the ED physician asking about smoking status was associated with increased perception of risk from smoking. Nevertheless, direct advice to quit smoking from either the patient's personal physician ("ever") or from the ED physician (this visit) was associated with significant increases in perceived risk from smoking.

It is imperative that future studies directed at smoking patients with respiratory illnesses. in the ED target ED physicians' understanding of the importance of providing direct advice to quit regardless of the chronicity of the patient's respiratory illness. Physicians were much less likely to provide advice to patients with lower respiratory illness, perhaps sharing the optimistic bias that the current illness was not associated with smoking. This perception is, of course, not true, and is not supported by the medical literature. Smoking patients who have not yet developed chronic respiratory illness and who quit smoking may be spared the long-term morbidity and inevitable mortality that those with chronic illness suffer. These patients will recognize the risk to their health from continued smoking and will be more ready to quit if the physician provides direct and clear advice. This study strongly supports future research that improves the probability that ED physicians' will appropriately address smoking with all of their patients who present with respiratory illness, regardless of chronicity.

## Conclusions

Results of this study indicate that direct advice from an ED physician significantly increases patients' perception of the health risks from smoking, and in turn, this perceived risk is strongly predictive of readiness to quit. Previous studies have shown that physician-delivered smoking cessation interventions, even when brief, can significantly increase smoking abstinence rates [51,52]. Although the ED may be an appropriate venue for preventive health interventions, there are numerous challenges to intervening in the ED setting. The scarcity of human resources, time pressures and focus on acute presenting problems make it particularly difficult to offer smoking cessation interventions. The use of physician extenders, such as paraprofessional health counselors, and or technological interventions (educational video) may help to address and overcome some of these barriers.

The emergency department is a venue in which to provide smoking cessation counseling. Smokers are over-represented among emergency department patients (with and without ARI) compared to population norms. The presence of respiratory symptoms such as wheezing or dyspnea focus the patient's attention on breathing and breathing-related issues. Patients seeking treatment for respiratory symptoms and illness may be perfectly placed to benefit from interventions that leverage respiratory symptoms and concerns to help motivate these patients to quit. Brief, physician-delivered interventions such as those described in the PHS guidelines and motivationally tailored interventions and treatments that incorporate biomarker feedback have both been shown to improve smoking cessation rates in health care settings. However, no data exist regarding the impact of smoking cessation interventions delivered in the ED to patients who present with Acute Respiratory Illness, a seemingly ideal "teachable moment." There is great opportunity for further research in this area. Broad application of effective smoking cessation interventions to respiratory patients in the ED has the potential to reach over 5 million smokers each year, and greatly decrease morbidity and mortality in this population of vulnerable smokers.

## Consent

All participants provided written informed consent to participate in this study prior to the collection of any data. Consent procedures, all written documents and procedures for handling subject data were reviewed and approved by the Human Subjects Review Board of the Miriam and Rhode Island Hospitals.

## Competing interests

The authors declare that they have no competing interests.

## Authors' contributions

BB participated in the design of the study, oversight of study conduct and statistical analyses, EJ participated in the conduct of the study and data analysis, BMB participated in the design of the study, weekly project meetings, PR participated in weekly project meetings and oversight of the day to day operations of the study, RN participated in study design. All authors participated in the writing and editing of the manuscript. All authors read and approved the final manuscript.

## Authors' information

Beth Bock, Ph.D., is an Associate Professor in the Department of Psychiatry and Human Behavior at Brown Medical School and works at the Centers for Behavioral and Preventive Medicine at the Miriam Hospital.

Dr. Bock's primary focus is the development of behavioral interventions for health behavior change in Emergency Medicine settings. Her research emphasizes the promotion of healthy lifestyles for the prevention of cardiovascular disease and cancer. Specific research projects include the examination of computer-based, tailored interventions for smoking cessation and exercise promotion.

Dr. Bock's recent research work includes two NIH-funded studies examining smoking cessation interventions among emergency medical patients. She is currently Principal Investigator on an NIH-funded study to develop a tobacco cessation intervention using text messaging. Dr. Bock has also received funding from NIH for a study examining the efficacy of tailored health communications for promoting exercise maintenance among cardiac rehabilitation patients. Dr. Bock is also working to develop tailored interventions to promote smoking cessation in pharmacy patients (funded by NIDA), and is working with QuitNet.com to develop and test a medication support system for website users (funded by NHLBI).
